# The Use of Endobronchial Ultrasound in the Diagnosis of Subacute Pulmonary Histoplasmosis

**DOI:** 10.1155/2015/510863

**Published:** 2015-10-12

**Authors:** Katarine von Lang Egressy, Mohammed Mohammed, J. Scott Ferguson

**Affiliations:** Division of Allergy, Pulmonary, and Critical Care Medicine, Department of Medicine, University of Wisconsin School of Medicine and Public Health, Madison, WI 53706, USA

## Abstract

*Objective*. Endobronchial ultrasound (EBUS) utility in diagnosis in malignant and granulomatous mediastinal disease has been well demonstrated. We propose to examine the role of EBUS transbronchial needle aspiration (EBUS-TBNA) in the diagnosis of subacute pulmonary histoplasmosis (SPH) with mediastinal lymphadenopathy in an area where histoplasmosis is endemic. *Methods*. A retrospective review was performed in a single academic institution between 2009 and 2012 of patients referred for EBUS-TBNA who had radiographic imaging and clinical symptomatology suspicious for SPH. Seven patients were reviewed. TBNA results showing granulomatous disease with areas of necrosis in the appropriate clinical setting were considered to be adequate for the diagnosis of SPH when alternative diagnosis was excluded. Patients underwent further clinical follow-up of 12 months to determine the final diagnosis. *Results*. All seven patients were felt to have SPH diagnosis reached by a combination of clinical presentation, EBUS-TBNA results, fungal serologies, and antigen testing. None of the patients needed further invasive procedures. *Conclusions*. EBUS-TBNA is a minimally invasive tool that can be used to support a diagnosis of SPH in patients with a high degree of clinical suspicion. EBUS-TBNA should be considered as an adjunctive diagnostic procedure for patients with SPH in an appropriate clinical setting.

## 1. Introduction

Histoplasmosis is the most common endemic fungal infection seen in humans. The Central River valleys in the Midwestern and South Central United States have endemicity for histoplasmosis. Approximately 250,000 individuals are infected annually but clinical manifestations of histoplasmosis occur in less than 5% [1]. Infections are sporadic, although large outbreaks of histoplasmosis may occur and histoplasmosis remains the most common cause for hospitalization amongst the endemic mycoses [2]. The morbidity and mortality of histoplasmosis are related to the duration and extent of systemic infection as well as underlying comorbidities. Around 1–5% may advance to subacute pulmonary histoplasmosis (SPH) [3].

Subacute pulmonary histoplasmosis (SPH) is defined by infection with* Histoplasma capsulatum* and the presence of symptoms for >1 month with a localized pulmonary opacity and/or hilar and mediastinal adenopathy. Clinical presentation may include protean symptoms of fevers, chills, malaise, weight loss, cough, and wheezing, symptoms that occur with multiple diseases. In addition, laboratory examination of serum, blood, and urine may result in wide spectrum of findings including anemia, leukocytosis, ESR and CRP elevation, and LFT abnormalities.

The diagnosis of histoplasmosis can be challenging. Cultures and identification of the organisms in tissue are often not helpful in SPH because of a low organism burden. Antigen detection is often variable in individuals with intact immune systems but can be useful if positive. Serology for histoplasmosis can be useful since the patient has often been infected for greater than one month allowing for a humoral immune response. High initial titers or rising titers over time are strongly suggestive of current or very recent infection, while a single low titer (<1 : 8) may suggest infections any time in the past [2].

Pulmonary physicians and thoracic surgeons are often asked to evaluate patients with hilar and mediastinal adenopathy. If SPH is suspected based on symptoms, the diagnosis may remain challenging because SPH unlike disseminated histoplasmosis tends to occur in immunocompetent individuals and presents with a variety of nonspecific symptoms that may be attributable to other causes [3].

EBUS-TBNA has emerged as an accurate, minimally invasive, safe technique for assessing undiagnosed mediastinal and hilar adenopathy, particularly in patients with lung cancer [4]. The use of EBUS-TBNA has not been reported for endemic fungal disease, although granulomatous inflammation has been reported especially in the evaluation of suspected sarcoidosis [5]. We hypothesized that EBUS-TBNA would provide additional confirmatory information for diagnosis in individuals with high suspicion of SPH in a geographical location with known endemic histoplasmosis and would complement the clinical exam in the elimination of other important causes of mediastinal and/or hilar adenopathy.

## 2. Methods

This study is a retrospective case review of seven patients referred to outpatient pulmonary clinic in a single academic institution for a workup of mediastinal/hilar lymphadenopathy. Inclusion criteria for our study consisted of hilar or mediastinal adenopathy with or without calcification, a clinical history suggestive of SPH and positive or indeterminate serology to* Histoplasma capsulatum.* In all seven patients a diagnosis of SPH was strongly considered prior to any invasive procedures based on epidemiologic, clinical, radiographic, and serological findings. Exclusion criteria consisted of the presence of an alternative diagnosis, immunocompromised state, or history of malignancy. On review of medical records between 2009 and 2012 in a single academic institution of patients referred to outpatient pulmonary clinic for workup of mediastinal/hilar adenopathy, seven patients were identified as meeting all the inclusion criteria and none of the exclusion criteria. Data was not collected on patients who did not meet inclusion criteria. All seven patients underwent a thorough clinical evaluation including urine and blood testing for fungal diseases and QuantiFERON TB Gold In-Tube test (Qiagen) and tuberculin skin testing for tuberculosis. Additional testing was performed as indicated by the clinical scenario. EBUS-TBNA was performed in all seven patients. Data were obtained from the procedure record, radiologic studies, pathology reports, clinical laboratory results, and clinic records. Institutional review board approval was obtained (UW–Madison IRB protocol number 2012-1164).

EBUS-TBNA was performed using a bronchoscope equipped with a distal 7.5 MHz linear ultrasound transducer probe (CP-EBUS, model XBF-UC260F; Olympus, Tokyo, Japan) and dedicated ultrasound image processor (model EU-ME1; Olympus). A dedicated aspiration needle (ViziShot, Olympus) was used to obtain specimens. The needle gauge, aspiration site, and the number of passes obtained [1, 2, 4] were performed at the discretion of the bronchoscopist. An on-site adequacy assessment was not available. A pair of slides was made for each aspirate: one was air-dried for Diff-Quick staining and one was fixed in 95% ethanol for Papanicolaou staining. Additional material was placed into saline for a cell block. All slides and the cell block material were examined by a cytopathologist. In cases where a suspicion for lymphoproliferative disease was present based on clinical or cytologic examination, additional specimens were sent for flow cytometry. In some cases, nodal aspirates were also sent for bacterial and fungal cultures. Subsequently, patients were followed clinically. Radiologic studies to confirm regression in lymphadenopathy were repeated at appropriate intervals for a period of up to 12 months.

The final diagnosis of SPH was based on combination of clinical and radiographic follow-up, serologic and antigen testing, cytologic findings, and the absence of an identifiable malignancy or other infections (e.g., tuberculosis).

## 3. Results

**Figure 1:**
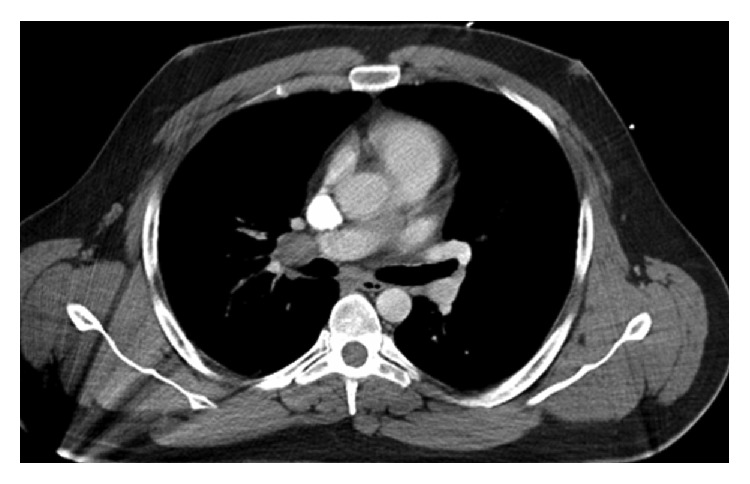
High resolution CT scan of hilar lymphadenopathy with pulmonary vein compression.

Basic demographic data and clinical characteristics of the seven subjects are presented in Table 1. The average age of our patient series was 39 with a range of 26–58 years of age. All had clinical symptoms persistent for more than one month consisting primarily of cough, fevers, malaise, and weight loss. One patient presented with hemoptysis. None of the patients had a previous history of underlying malignancy or current infectious disease. Only one patient had an identifiable exposure to histoplasmosis while remodeling an old house that was contaminated with heavy bird droppings. Radiographic images showed mediastinal and/or hilar lymphadenopathy in seven out of seven patients, small pulmonary subcentimeter nodules in two out of seven patients, and pulmonary vein compression in one patient presumed due to postinflammatory fibrosis (Figure 1).

**Figure 2:**
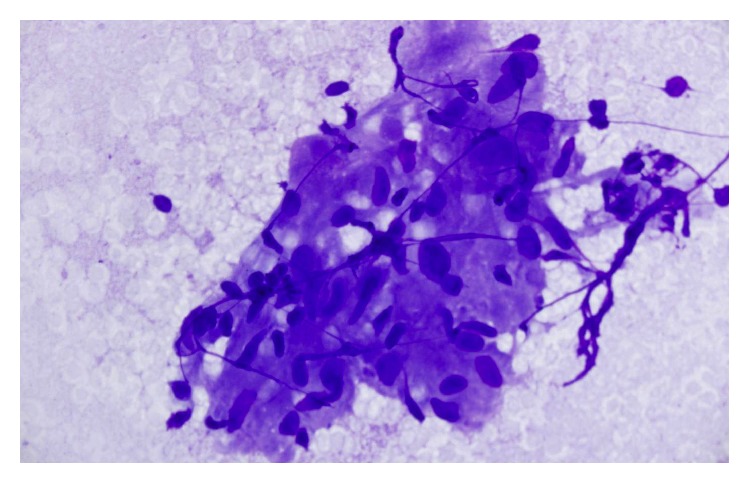
Diff-Quik stain of lymph node tissue with loosely organized granuloma.

All patients had EBUS detectable mediastinal or hilar lymphadenopathy. Lymph node size measured from 15 mm to 35 mm in the short axis dimension (average 22 mm). Loosely organized granulomatous inflammation with areas of necrosis was present in five out of seven patients (Figure 2). Two patients had inadequate sampling. No fungal elements were identified in any of the patients' specimens.

All patients underwent urine and serum histoplasma and blastomycosis antigen testing, complement fixing antibody for histoplasma mycele and yeast forms, and blastomycosis antibody (Table 2). All patients had positive (>1 : 8 by complement fixation) serology for histoplasmosis. One patient had antigenemia and antigenuria and one patient had antigenuria alone. One patient had both positive histoplasma and blastomyces urinary antigens however due to high cross reactivity reactions of these fungi (as high as 90%) [6] as well as clinical picture; the patient was felt to have infectious process consistent with SPH. In one patient (case number 1), nodal material was sent for flow cytometry, which was negative for a monoclonal population of lymphocytes.

Four of the patients (cases 1, 3, 4, and 5) were treated with itraconazole for persistent or worsening symptoms. Infectious Disease Society of America guidelines were followed [7]. Clinical and radiographic follow-up was performed in all patients for up to 12 months. All patients in the series had improvement in symptoms (resolution of cough, fevers, and sweats) and radiographic findings (decreased nodal size and decreased nodules if present) over a 3-to-12-month period. None of the treated patients required retreatment, and none of the seven patients went on to further invasive procedures such as mediastinoscopy or video-assisted thoracoscopic surgery.

## 4. Discussion

Indeterminate mediastinal and hilar adenopathy in patients with nonspecific symptoms is often a clinical challenge for the treating pulmonologist or thoracic surgeon. In selected patients with a high index of clinical suspicion, indeterminate or weakly positive serology for histoplasmosis and cytologic confirmation of granulomatous inflammation may reduce the need for further invasive testing. This study demonstrates that EBUS-TBNA can be used in the evaluation of mediastinal and hilar adenopathy when histoplasmosis is suspected. Although our study is limited by number and nature of a retrospective review, our data show that mediastinal and hilar lymph nodes in the setting of suspected histoplasmosis are evaluable by EBUS and that cytologic findings are consistent with this diagnosis in most cases. Further, culture of nodal material obtained by EBUS-TBNA was negative for histoplasmosis in all cases. None of the patients were diagnosed with an alternative disease, even after careful radiographic and clinical follow-up.

The diagnosis of SPH in this study was made using a combination of clinical suspicion along with focused laboratory testing, the absence of alternative diseases, and follow-up. We feel that this is a valid model in an area where histoplasmosis is endemic. In other parts of the USA and world, practitioners would have to consider the epidemiology of the endemic mycoses for their region. The most frequently cited study that describes areas of endemicity for histoplasmosis in the United States was published in 1969 by Edwards et al. The study identified histoplasmosis endemicity on the basis of histoplasma skin testing, a diagnostic method of unknown sensitivity and probably poor specificity [8].

In the United States, an estimated 60% to 90% of people who live in areas surrounding the Ohio and Mississippi River Valleys (where* Histoplasma* is common in the environment) have been exposed to the fungus at some point during their lifetime [9]. One study calculated the incidence of histoplasmosis in adults aged 65 years and older in the USA to be 3.4 cases per 100,000 population. Rates were highest in the Midwest, with estimated 6.1 cases per 100,000 population [10].

Although findings of EBUS-TBNA were consistent with SPH, they were not confirmatory (no fungal elements observed in any specimen and negative fungal cultures). Pathologic findings in SPH may overlap or closely resemble such disease states as sarcoidosis or tuberculosis as well as malignancy in some cases, and interpretation of pathology may be very challenging as observation of fungal organisms in aspirate smears is unusual [11, 12]. Accordingly, serological workup and clinical/radiographic data and follow-up played an important role in reaching the final diagnosis in all seven patients.

In this selected patient population where the risk of SPH was considered to be much higher than the risk of malignancy, cytopathology obtained by EBUS-TBNA combined with additional testing was considered adequate and no further invasive testing was performed. However, in a patient population where the pretest probability for malignancy is higher, additional invasive testing would likely be warranted depending on the clinical circumstances. Additionally, other causes of granulomatous inflammation (e.g., metastatic cancers, lymphoma, and sarcoidosis) should always be carefully considered based on the clinical situation. A retrospective review of patients referred for mediastinal lymph node sampling in a large academic center demonstrated that despite presence of mediastinal calcification (56%) and lymph nodal enlargement in an area with known endemic histoplasmosis, EBUS-TBNA nodal sampling was not affected, and positive and negative predictive values of this test were comparable to other previously published studies [13].

Current recommendations for the diagnosis of* Histoplasma capsulatum* infection include a combination of several different modalities to achieve the highest yield depending on the presenting symptoms and timing of the exposure [5]. In SPH, the diagnosis can be challenging as most often these patients have nonspecific symptoms and findings that have a broad differential diagnosis. Although >90% of histoplasmosis infections are asymptomatic, mediastinal/hilar lymphadenopathy and associated nonspecific symptoms may be present in 5–10% of immunocompetent patients [2]. Currently, testing for antibodies by immunodiffusion or complement fixation technique is considered more sensitive for SPH than tests for an antigen, as fungal burden is low and pulmonary infection is often very localized in immunocompetent patients [3].

There are significant limitations of this study. First, this is a retrospective review of selected patient group who were strongly suspected of having SPH. As such, there is an intentional selection bias, and there are variables that we do not have control over, such as the skill of the bronchoscopist, the skill of the cytologist, and the exact methods used to perform diagnostic testing. Therefore, we are not able to conclude that EBUS-TBNA has a high utility in discriminating one cause of adenopathy from another. However we can conclude that, in this patient population, EBUS-TBNA should result in granulomatous inflammation in most cases.

Prospective studies with greater number of patients as well as more controlled data acquisition and uniform follow-up that include this population and others may be able to define the discriminant function of EBUS-TBNA in SPH. However, the precise incidence of SPH is not known [9]. These seven cases were accumulated over the course of 3 years in a busy academic pulmonary clinic. The likelihood that a prospective trial of EBUS for SPH could be accomplished is low without collaborations from multiple institutions.

Furthermore, 2 of 7 patients had inadequate sampling. Unfortunately this study is too small for us to determine if a value of 2 of 7 is significant. No currently available studies directly address inadequate sampling of EBUS-TBNA in patients with suspected infections. Inadequate sampling or nondiagnostic aspirates occur with variable frequency [14] and are potentially related to needle size, cytology procedures, operator technique, the specific target disease, and the prevalence of the disease in question [15]. Further study will be necessary to answer these questions. In the two patients who had inadequate samples by EBUS-TBNA, one had high urinary antigen positivity and the other had rising titers of antibody to histoplasmosis. Therefore, we feel that both of these individuals had SPH.

The body of literature accumulates on the use of EBUS-TBNA in diagnosis of mediastinal lymphoma [16], tuberculosis, sarcoidosis [5], and malignancy [17]. Although diagnosis of lymphoma by EBUS-TBNA technique remains a subject of controversy due to the small available retrospective studies as well as a minimal tissue yield that is available for pathologic analysis by EBUS-TBNA, future advances such as the use of miniforceps may improve the diagnostic yield and establish EBUS-TBNA as an initial procedure of choice [18].

Our study provides evidence that EBUS may be helpful in the evaluation of infectious diseases as well. We believe that in situations where there is a high suspicion of SPH (clinical symptoms, serology), EBUS-TBNA is useful in the elimination of alternative diseases and can provide compatible pathologic findings. If a presumptive diagnosis of SPH is made by a combination of clinical signs, serology, and EBUS, patients should be followed for response to treatment or resolution of the clinical findings. Further prospective studies are anticipated that will define the utility and efficacy of EBUS-TBNA in the evaluation of patients with mediastinal/hilar adenopathy and a high likelihood of infection.

## 5. Conclusion

The use of EBUS-TBNA is becoming widespread in diagnosis and staging of lung malignancies. No current literature exists on the role of EBUS-TBNA in infectious causes of mediastinal/hilar lymphadenopathy. This study demonstrates that EBUS-TBNA is a minimally invasive adjunctive procedure that can be used in patients in whom clinical, radiographic, and serological data raise a high index of suspicion for SPH. Furthermore, EBUS-TBNA with clinical and radiographic follow-up appears to have avoided additional invasive diagnostic testing. Further studies are needed to examine the efficacy and utility of EBUS-TBNA in patients with suspected infections and presence of mediastinal/hilar adenopathy.

## Figures and Tables

**Table 1:** Patient demographics and clinical features.

Case number	Age	Sex	Smoking Hx	Probable exposure to *Histoplasma capsulatum*	Clinical symptoms	Symptom duration	Radiographic findings
1	26	Male	None	Significant	Low grade fevers, cough, weight loss	4 months	Bilateral hilar and mediastinal adenopathy

2	30	Male	10 ppy	Minimal	fatigue	3 months	Right hilar adenopathy

3	50	Male	None	Minimal	Fevers, night sweats, cough, weight loss	1 year	Mediastinal and hilar adenopathy, small bilateral pulmonary nodules

4	34	Female	None	Minimal	Fevers, malaise, night sweats	9 months	Mediastinal and hilar adenopathy, small bilateral pulmonary nodules

5	57	Female	5 ppy	Minimal	Cough, fevers	8 months	Mediastinal adenopathy, small bilateral pulmonary nodules

6	36	Male	20 ppy	Minimal	Recurrent hemoptysis	3 months	Mediastinal and hilar adenopathy

7	29	Male	None	Minimal	Cough, fevers, fatigue	5 months	Left hilar adenopathy

**Table 2:** Diagnostic results.

Case number	Lymph node size	Stations sampled	Cultures	Infectious serologies	Histoplasmosis antigen	Cytology findings
1	35 mm	7	Negative	Acute 1 : 16 Convalescent 1 : 64	Negative	Loosely organized granuloma with necrotic debris

2	15 mm	11 R	Negative	Acute 1 : 32	Negative	Loosely organized granuloma with necrotic debris

3	15 mm	11 L	Negative	Acute 1 : 128	Positive urine Positive serum	Loosely organized granuloma with necrotic debris

4	25 mm	10 L	Negative	Acute 1 : 32	Positive urine	Nondiagnostic

5	20 mm	7	Negative	Acute 1 : 8 Convalescent 1 : 64	Negative	Nondiagnostic

6	30 mm	7	Negative	Acute 1 : 16	Negative	Loosely organized granuloma with necrotic debris

7	23 mm	11 L	Negative	Acute 1 : 16 Convalescent 1 : 512	Negative	Loosely organized granuloma with necrotic debris
